# Genetic investigation of patients with autosomal recessive ataxia and identification of two novel variants in the *SQSTM1* and *SYNE1* genes

**DOI:** 10.1038/s41439-024-00292-x

**Published:** 2024-08-30

**Authors:** Diana Mokhtari, Mohammad Jahanpanah, Nasim Jabbari, Hamed Azari, Sana Davarnia, Haleh Mokaber, Sara Arish, Rasol Molatefi, Vahid Abbasi, Behzad Davarnia

**Affiliations:** 1 Department of Genetics and Pathology, Ardabil University of Medical Sciences, Ardabil, Iran; 2 Department of Animal Biology, Faculty of Natural Science, University of Tabriz, Tabriz, Iran; 3 Tabriz University of Medical Sciences, Tabriz, Iran; 4 Department of Biology, Ardabil Branch, Islamic Azad University, Ardabil, Iran; 5 Department of Pediatrics, Bo-Ali Children’s Hospital of Ardabil University of Medical Sciences, Ardabil, Iran; 6 Cancer Immunology and Immunotherapy Research Center, Ardabil University of Medical Sciences, Ardabil, Iran; 7 Department of Neurology, Ardabil University of Medical Sciences, Ardabil, Iran

**Keywords:** Medical genetics, Neurodegeneration

## Abstract

Hereditary ataxias are classified by inheritance patterns into autosomal dominant, autosomal recessive, X-linked, and mitochondrial modes of inheritance. A large group of adult hereditary ataxias have autosomal dominant inheritance, and autosomal recessive cerebellar ataxias (ARCAs) are rare, with greater diversity in phenotypic and genotypic features. Therefore, comprehensive genetic testing is useful for identifying the genes responsible for ARCAs. We identified two novel pathogenic variants of the SQSTM1 and SYNE1 genes via whole-exome sequencing in patients with ARCAs.

Hereditary ataxias are classified into autosomal dominant, autosomal recessive, X-linked, and maternal inheritance types on the basis of their type of inheritance^1^. The diagnosis of hereditary ataxia can be established on the basis of typical neurological findings, a positive family history, and the exclusion of nongenetic ataxias^1^. Autosomal recessive cerebellar ataxias (ARCAs) are complex neurodegenerative disorders with highly diverse phenotypes and genotypes^2^. ARCAs are considered to be early-onset^3^, unlike the autosomal dominant types, among which the age of onset overlaps^1^. Various genes have been reported to be responsible for different subtypes of ARCAs, with Friedreich’s ataxia (*FRDA* gene) and ataxia telangiectasia (*ATM* gene) being the most common^4^. Although most ARCAs can be distinguished by unique clinical features, definitive diagnosis is challenging because of overlapping phenotypes^5^. Therefore, it is necessary to use genetic diagnostic methods to identify the different genes responsible for various ARCAs. Over 90 genes have been reported to be associated with ARCAs^6^.

Compared with traditional techniques, next-generation DNA sequencing (NGS) techniques are very powerful, affordable, and fast diagnostic tools for determining the genetic causes of neurological disorders such as ataxia^7^. Recognizing gene defects is a major step in detecting different molecular pathways and is an important prerequisite for the development of effective targeted molecular therapies^8^. Therefore, this study aimed to identify the genes and mutations associated with autosomal recessive ataxia in two families with consanguineous marriages in Iran. In this study, owing to the diverse phenotypic and genotypic features of patients with ARCAs, whole-exome sequencing (WES, an NGS-based test) was used as an appropriate method for identifying the genetic basis of Mendelian disorders of unknown etiology^9,10^.

This study was approved by the Ethics Committee of the Ardabil University of Medical Sciences (IR.ARUMS.REC.1402.019), and written consent was obtained from the parents. All parents were phenotypically normal. Pedigrees were drawn, and the patients underwent neurological examinations, laboratory tests, and MRIs. Nongenetic causes were ruled out. A peripheral blood sample was obtained, high-quality DNA was extracted via the standard salting-out method, and sample preparation was performed. An Agilent Human All Exon Kit was used for exome enrichment of total genomic DNA, and paired-end sequencing was performed via an Illumina HiSeq sequencer. The functional annotation of identified variants was performed via the ANNOVAR tool. Neutral and previously reported variants with a frequency of 1% or above were filtered out from further analysis. Whole-exome sequencing revealed two novel variants in the *SQSTM1* and *SYNE1* genes.

The proband in family I is a 50-year-old female with ataxia who was born in a family with consanguineous marriage (Fig. 1a). The birth measurements were normal. She started to sit, crawl, and walk on time and had normal development until 7 years of age. Symptoms started with speech delay at the age of 7 and a few months later with unsteady gait and ataxia. She has experienced cognitive decline; however, it was categorized as mild. Currently, the patient can walk short distances and eat food without assistance. She follows orders and can communicate with words and small phrases. Vertical gaze palsy is present, but dystonia was not detected. She has not experienced any seizures; however, her brother suffers from intellectual disability and epilepsy with no ataxia. Brain MRI revealed mild cerebellar atrophy.

**Fig. 1:**
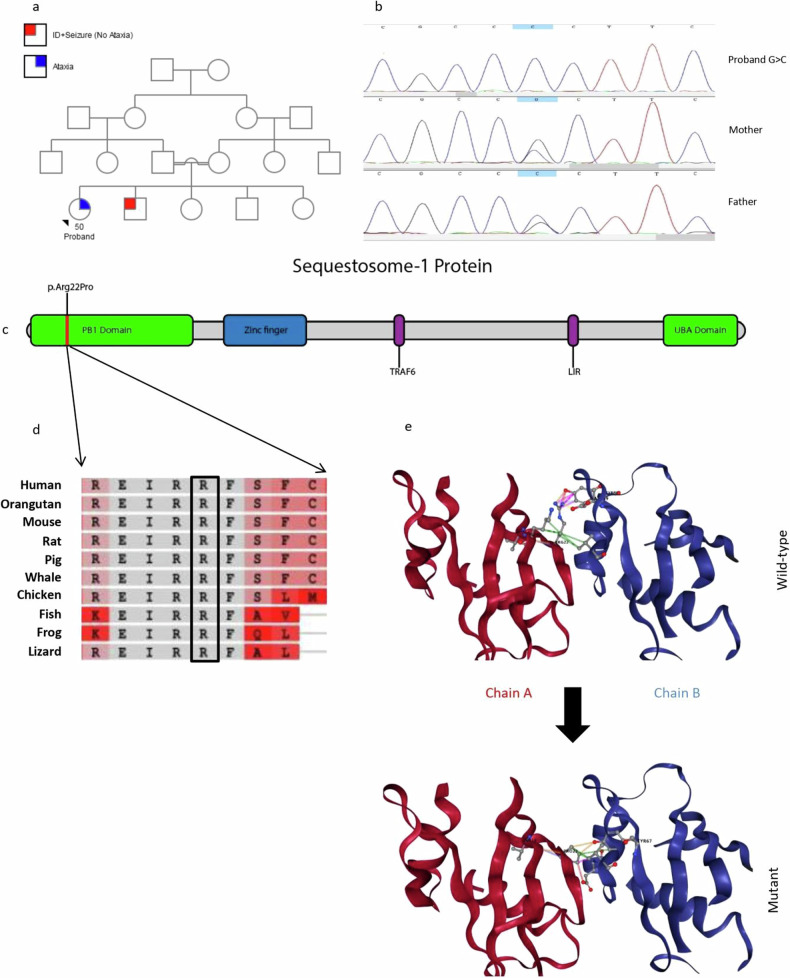
The pedigree, molecular analysis and predicted structure of the mutant protein in the patient in family I. **a** Pedigree of the proband in Family I. **b** Sequencing results of Family I (*SQSTM1* gene). **c** Schematic diagram of the sequestosome-1 protein and location of the p.Arg22Pro mutation. **d** Evolutionary conservation analysis of arginine amino acids in different species (human: *Homo sapiens*, Orangutan: *Pongo abelii*, mouse: *Mus musculus*, rat: *Rattus norvegicus*, Pig: *Sus scrofa*, whale: *Physeter microcephalus*, chicken: *Gallus gallus*, fish: *Gadus morhua*, frog: *Xenopus laevis*, lizard: *Anolis carolinensis*), **e** predicted structure of wild-type and mutant proteins via the DynaMut-2 web server.

The WES results revealed that the proband was homozygous for (NM_003900.5) c.65G > C, p.Arg22Pro in the *SQSTM1* gene (Fig. 1b, c). This mutation replaces the amino acid arginine with proline. Conservation analysis revealed that the arginine amino acid at this position is highly conserved across ten different species (Fig. 1d). The DynaMut2 web server was used to predict the structure of the wild-type and mutant proteins (Fig. 1c). On the basis of the ACMG guidelines, this variant was categorized as “likely pathogenic” (PM1, PM2, PP3 and PP4). Pathogenic mutations in the *SQSTM1* gene are associated with childhood-onset neurodegeneration with ataxia, dystonia, and gaze palsy (NADGP) (OMIM: 617145). This phenotypic spectrum was first defined by Haack et al. in 2016. Previously reported pathogenic variants and their clinical manifestations are listed in Table S1.

The proband in Family II is a 36-year-old woman with ataxia in a family with a consanguineous marriage (Fig. 2a). She was born via normal vaginal delivery. Her sitting, walking, and growth were normal, and she had a normal childhood. The first symptoms started at the age of 25, with difficulty maintaining balance and respiratory distress, and her symptoms worsened over time. Currently, the proband has spasticity of the lower limbs, muscle atrophy, difficulty breathing, and dysarthria. She cannot move or stand without assistance. She can say words and small phrases but not full sentences. Visual symptoms were not present. Brain magnetic resonance imaging (MRI) revealed cerebellar atrophy, and spine and neck MRI results revealed decreased neck lordosis. She has two brothers and one sister with identical symptoms that began in the third decade of their life, and two of them are deceased.

**Fig. 2:**
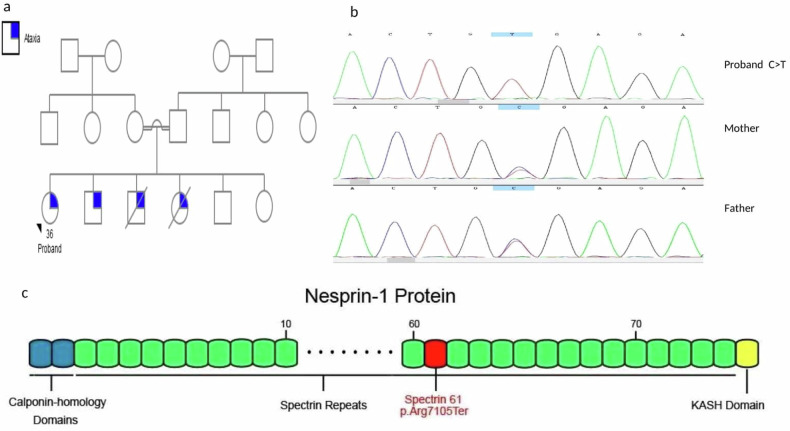
The pedigree and the molecular analysis of the patient in family II. **a** Pedigree of the proband in Family II. **b** Sequencing results for family II (*SYNE1* gene). **c** Schematic diagram of the nesprin-1 protein and location of the p.Arg7105Ter mutation.

WES revealed a novel homozygous mutation of cytosine to thymine (NM_182961.4): c.21313C > T, p.Arg7105Ter in the *SYNE1* gene (Fig. 2b, c), creating a premature stop codon. On the basis of the ACMG guidelines, the Franklin website classified this variant as “pathogenic” (PVS1, PM2 and PP4). Pathogenic mutations in *SYNE1* are associated with spinocerebellar ataxia, autosomal recessive 8 (SCAR8) (OMIM: 610743).

Childhood-onset neurodegeneration with ataxia, dystonia, and gaze palsy (NADGP) is a progressive disorder with three characteristics: gait ataxia, cognitive decline, and gaze palsy^11^. Studies have shown that symptoms usually start between 6 and 15 years of age with ataxia, but in this case, it started with dysarthria. Vertical gaze palsy and dystonia are reported to be common manifestations in these patients^11,12^; however, dystonia has been reported in only 50% of the cases thus far, and in the current case, dystonia was not detected.

*SQSTM1* is located on chromosome 5q35.3 and synthesizes an NM_003900.5 mRNA with 8 exons, which is responsible for synthesizing the sequence-1 protein with 440 amino acids^13,14^. This protein is responsible for modulating enzyme function through several domain interactions and has an essential role in the degradation of protein molecules^15^. The PB1 domain is necessary for localization into ubiquitin-containing inclusion bodies and interacts with many other proteins^16,17^. Recently, it has been shown that the PB1 domain is essential for the formation of stable large multiprotein complexes^18^. Previous studies have shown that neurodegeneration can be due to defects in autophagy, mitochondrial function, and ubiquitin–proteasomal degradation pathways^19^. We identified a homozygous mutation, NM_003900.5: c.65G > C, p.Arg22Pro, in the *SQSTM1* gene. This mutation replaces the arginine amino acid with proline and is located in the PB1 domain of this protein. The DynaMut2 web server predicted this mutation to be destabilizing (predicted stability change = ΔΔG −0.43 kcal/mol). The arginine amino acid in the wild-type protein has a greater interaction frequency with chain B than the proline amino acid in the mutant protein does, and this change could destabilize the protein structure and change its function. This variant was identified for the first time.

Autosomal recessive spinocerebellar ataxia-8 (SCAR8) is an autosomal recessive and neurodegenerative disorder that often has late onset and slow progression and was first identified in individuals from Beauce in Quebec^20,21^. This ataxia is usually progressive cerebellar ataxia with rare and mild extracerebellar symptoms in adulthood and leads to gait ataxia, lower and upper motor neuron disease, brainstem dysfunction, and musculoskeletal abnormalities^20^. Recent studies have suggested that *SYNE1* ataxia is a complex multisystemic syndrome that is common worldwide^22^. In our case, the proband in family II had typical symptoms similar to those of previously reported patients, in addition to the loss of cervical lordosis. There were four individuals in this family with ataxia, and only one of them was genetically assessed; all four had late-onset ataxia.

*SYNE1*, located on chromosome 6q25.2 and containing 146 exons, is an enormous gene that encodes a 27,708-bp long mRNA, NM_182961.4, that is converted into a protein with 8797 amino acids called nesprin-1^19,20^. As a structural protein of spectrin, this protein is involved in connecting the plasma membrane to the actin cytoskeleton^23^. Nesprin-1 is expressed in different tissues and significantly expressed in striated muscles and the cerebellum^24^. The molecular etiology of these symptoms is not fully understood. However, Razafsky et al. proposed that the pathogenicity of these mutations originates from dysfunctions of KASH-LESS variants of the giant Nesprin1 isoform, which is specifically expressed in the central nervous system^25^. Our proband was homozygous for the pathogenic variant NM_182961.4:c.21313C > T, p.Arg7105Ter in exon 116 of *SYNE1*, which creates a premature stop codon. Previous studies have shown that nonsense and frameshift mutations in the *SYNE1* gene lead to nonsense-mediated decay of the mRNA^20,21^. This variant was identified for the first time.

## Supplementary information

Supplementary file

## Data Availability

The relevant data from this Data Report are hosted at the Human Genome Variation Database at 10.6084/m9.figshare.hgv.3433; 10.6084/m9.figshare.hgv.3436.
