# Supplementary material for: Genetic investigation of patients with autosomal recessive ataxia and identification of two novel variants in the SQSTM1 and SYNE1 genes
Source: Hum Genome Var. 2024 Aug 30;11:35. doi: 10.1038/s41439-024-00292-x (PMC11364807; doi:10.1038/s41439-024-00292-x)
Supplement: Supplementary file 1 — Supplementary file [file 41439_2024_292_MOESM1_ESM.docx]

| Author | Onset | Gender | Ethnicity | Consanguinity | Variants | Ataxia | Dysarthria | Cognitive Decline | Gaze palsy | Dystonia |
| --- | --- | --- | --- | --- | --- | --- | --- | --- | --- | --- |
| Haack et al. | 7-15 | 7F-2M | German (3)  Emirati (3)  Kurdish (2)  Finnish (1) | 2/9 | c.2T>A(3)  c.311_312del(3)  c.286C>T(3) | 9/9 | 9/9 | 8/9 | 7/9 | 7/9 |
| Muto et al. | 6-12 | 4F-7M | Iranian (9)  Italian (2) | 11/11 | c.875_876insT (7)  c.934_936delinsTGA(2)  c.301+2T>A (2) | 11/11 | 11/11 | 11/11 | 11/11 | 2/11 |
| Ramirez et al. | 8-14 | 1F-3M | Mexican (2)  Jordanian (2) | 2/4 | c.257_259delins35 &  c.301+1G>T (2)(CH)  c.823_824delAG (2) | 4/4 | 4/4 | 4/4 | 2/4 | 4/4 |
| Vedartham et al. | 8 | F | Indian | 1/1 | c.712_713insTCCTCCGAGTGTGAATTTCCTGA | 1/1 | 1/1 | 0/1 | 1/1 | 0/1 |
| Akkari et al. | 9 | F | Tunisian | 0/1 | c.823_824delAG | 1/1 | 1/1 | 1/1 | 1/1 | 1/1 |
| Kilic et al. | 6 | M | Turkish | 1/1 | c.55G>T | 1/1 | 1/1 | 1/1 | 1/1 | 0/1 |
| Jalali et al. | NA | F | Iranian | 1/1 | c.790delA | 1/1 | 1/1 | 1/1 | 1/1 | 1/1 |
| Salari et al. | 7 | M | Iranian | 1/1 | c.838G>T | 1/1 | 1/1 | 1/1 | 1/1 | 1/1 |
| This study | 7 | F | Iranian | 1/1 | c.65G>C | 1/1 | 1/1 | 1/1 | 1/1 | 0/1 |
| Total | 6-15 | 16F-14M | - | 20/30 | - | 30/30 | 30/30 | 28/30 | 26/30 | 16/30 |

Table S1. Summary of clinical features of previously reported variants in *SQSTM1* gene. F: Female, M: Male, NA: Not Available,

CH: Compound Heterozygous

**References**

1. Haack TB, Ignatius E, Calvo-Garrido J, Iuso A, Isohanni P, Maffezzini C, Lönnqvist T, Suomalainen A, Gorza M, Kremer LS, Graf E. Absence of the autophagy adaptor SQSTM1/p62 causes childhood-onset neurodegeneration with ataxia, dystonia, and gaze palsy. The American Journal of Human Genetics. 2016 Sep 1;99(3):735-43.
2. Muto V, Flex E, Kupchinsky Z, Primiano G, Galehdari H, Dehghani M, Cecchetti S, Carpentieri G, Rizza T, Mazaheri N, Sedaghat A. Biallelic SQSTM1 mutations in early-onset, variably progressive neurodegeneration. Neurology. 2018 Jul 24;91(4):e319-30.
3. Zúñiga-Ramírez C, de Oliveira LM, Kramis-Hollands M, Algarni M, Soto-Escageda A, Sáenz-Farret M, et al. Beyond dystonia and ataxia: expanding the phenotype of SQSTM1 mutations. Parkinsonism & Related Disorders. 2019 May 1;62:192-5.
4. Vedartham V, Sundaram S, Nair SS, Ganapathy A, Mannan A, Menon R. Homozygous sequestosome 1 (SQSTM1) mutation: a rare cause for childhood-onset progressive cerebellar ataxia with vertical gaze palsy. Ophthalmic Genetics. 2019 Jul 4;40(4):376-9.
5. Akkari M, Kraoua I, Klaa H, Benrhouma H, Ben Younes T, Rouissi A, Chaabouni M, Ben Youssef‐Turki I. SQSTM1 mutation: description of the first Tunisian case and literature review. Molecular Genetics & Genomic Medicine. 2020 Dec;8(12):e1543.
6. Kilic MA, Kipoglu O, Coskun O, Karacabey BN, Yesilyurt A, Yildiz EPet al. Homozygous SQSTM1 nonsense variant identified in a patient with brainstem involvement. Brain and Development. 2021 Nov 1;43(10):1039-43.
7. Jalali H, Khoshaeen A, Mahdavi MR, Mahdavi M. First report of novel mutation (c. 790del) on SQSTM1 gene on a family with childhood onset of progressive cerebellar ataxia with vertical gaze palsy. Clinical Case Reports. 2022 Aug;10(8):e6203.
8. Salari M, Etemadifar M, Neshat Ghalibaf M, Azizi F, Davoodi M, Asadi S. Neurodegeneration, ataxia, dystonia, and gaze palsy (NADGP) syndrome with nocturnal paroxysmal head tremor. Movement Disorders Clinical Practice. 2023 Apr 20.
